# Revival of the mother-baby friendly initiative (MBFI) in South Africa: towards a quality improvement project

**DOI:** 10.1186/s13006-024-00634-z

**Published:** 2024-04-22

**Authors:** Welma Lubbe, Zandile Kubeka, Ann Behr, Gilbert Tshitaudzi, Sithembile Dlamini-Nqeketo, Jessica Botha

**Affiliations:** 1 NuMIQ Research Focus Area, North-West University, Potchefstroom, South Africa; 2 National Department of Health, Pretoria, South Africa; 3 National Department of Health, Pretoria, South Africa; 4 United Nations Children’s Fund, Pretoria, South Africa; 5 World Health Organization South Africa, Pretoria, South Africa

**Keywords:** Breastfeeding, Mother-Baby friendly initiative, Quality improvement project, Revitalisation, South Africa

## Abstract

**Background:**

The discontinuation of “Mother-Baby Friendly” accreditation, coupled with the impact of the COVID-19 pandemic, has contributed to reduced breastfeeding rates observed in parts of South Africa. Consequently, the Child, Youth and School Health cluster of the National Department of Health, with support from the World Health Organization and United Nations Children’s Fund, organised a Mother-Baby Friendly initiative revitalisation workshop.

**Methods:**

Held in Johannesburg, South Africa, on June 29–30, 2022, the workshop brought together local and international breastfeeding promotion experts to engage on issues related to the revitalisation of the Mother-Baby Friendly Initiative. The workshop included presentations and group sessions aimed at setting expectations, evaluating the Ten Steps to Successful Breastfeeding, and developing actionable revitalisation strategies.

**Results:**

Inadequate monitoring of the Mother-Baby Friendly Initiative implementation and adherence to the Ten Steps was identified as a major implementation bottleneck. Participants identified steps ten (coordinating discharge so that parents and their infants have timely access to ongoing support and care), five (supporting mothers to initiate and maintain breastfeeding and manage common difficulties), and two (ensuring that staff have sufficient knowledge, competence, and skills to support breastfeeding) of the Baby-Friendly Hospital Initiative as the most difficult to implement. Step seven (enabling mothers and their infants to remain together and to practise rooming-in 24 h a day) was the least difficult to implement. Workshop participants identified the following proposed solutions to revitalise breastfeeding promotion: strengthening capacity building and mentorship, improving monitoring and accountability measures, and certification of facilities meeting the initiative’s standards.

**Conclusion:**

Current breastfeeding policies and practices must be evaluated by the National Department of Health in collaboration with provincial and private representatives of the initiative to effectively revitalise the Mother-Baby Friendly Initiative. Moreover, an integrative monitoring framework must be developed through stakeholder engagement, role clarification, and ownership. While collaboration between the private and public sectors is required to promote training and communication within healthcare facilities and communities.

## Background

In response to the Innocenti Declaration of 1990, the United Nations Children’s Fund (UNICEF) and the World Health Organization (WHO) established the Baby-Friendly Hospital Initiative (BFHI) in 1991 [1] (Table 1), with the goal of encouraging healthcare providers to improve facility practices that promote, support and protect breastfeeding [2]. In 2018, the WHO issued a revised version of the BFHI Ten Steps, which were to be implemented as a standard of care in all healthcare facilities with maternity beds to create an enabling environment for the early initiation and maintenance of breastfeeding [3]. The revised WHO/UNICEF BFHI implementation guidance emphasises the importance of fully integrating breastfeeding protection, promotion, and support into the healthcare system, including both private and public facilities [3]. Moreover, monitoring facility adherence to the Ten Steps is a critical component of the revised guideline to improve healthcare quality for mothers and newborns [3].

**Table 1:** Timeline of selected strategies and policies to support and protect breastfeeding in South Africa

1990	Innocenti Declaration
1991	UNICEF and WHO establish the BFHI [1]
1994	SA’s first baby-friendly hospital declared
2001	Strategy for the Implementation of the Baby Friendly Hospital Initiative in South Africa, 2001–2004 [7]
2008	Review of the implementation of the Baby Friendly Hospital Initiative in Public Maternity Units in South Africa [8]
2011	Tshwane Declaration of Support for Breastfeeding [9]
2011	Renaming of the BFHI to the Mother-Baby Friendly Initiative (MBFI) in SA
2018	UNICEF/WHO “Protecting, promoting and supporting breastfeeding in facilities providing maternity and newborn services: the revised Baby-Friendly Hospital Initiative” Implementation Guidance [3]

### The BFHI in South Africa

St. Monica’s Maternity Hospital in the Western Cape was declared South Africa’s (SA) first baby-friendly facility in 1994 [4]. The National Department of Health (NDoH) steered the BFHI. However, it relied heavily upon external trainers and assessors. The provision of training materials for maternity staff and trainers of the 18-hour course ‘Breastfeeding Promotion and Support in a Baby-Friendly Hospital’ and subsequent 20-hour course was significantly facilitated by the WHO and UNICEF [5]. In addition to providing tools to facilitate self-evaluation and monitoring as well as external assessment of hospitals [5], these organisations, as well as the International Baby Foods Action Network (IBFAN) provided a great deal of support in implementing the BFHI in SA [6]. In 2001, a BFHI implementation strategy was developed to increase the number of accredited public facilities [7]. Later in 2008, the SA NDoH and UNICEF commissioned a BFHI review led by the University of the Western Cape to evaluate the implementation of the BFHI in public maternity facilities [8]. Results strongly suggested that BFHI-accredited facilities implemented criteria better, however, the principles of the guideline transformed all facilities, including those working toward accreditation [8]. Accredited facilities which had lower maternity staff rotation rates, active BFHI committees, and better integration of programmes such as kangaroo mother care (KMC) and prevention of mother-to-child transmission (PMTCT) were shown to perform better overall [8].

In 2011, SA adopted the Tshwane Declaration of Support for Breastfeeding [9], and the country renamed the BFHI to the Mother-Baby Friendly Initiative (MBFI) to include mothers and communities [10]. The *Ten Steps* of the BFHI were modified for MBFI implementation, and a national plan outlined the role of government, the sharing of resources and accountability at all levels. The plan sought to ensure managers and stakeholders were sensitised to incorporate infant feeding goals and objectives into existing policies and programmes.

### Impact of the MBFI in South Africa

The MBFI has the potential to significantly improve breastfeeding practices in SA, particularly the early initiation of breastfeeding and the elimination of practices that hinder breastfeeding [11]. Following the initial implementation of the BFHI, the 2003 SA Demographic and Health Survey (SADHS) found that 61% of mothers initiated breastfeeding within an hour of birth, compared with 45% in 1998 [12]. The 2008 South African National HIV Prevalence, Incidence, Behaviour and Communication Survey found that more than 80% of mothers initiated breastfeeding within one hour after birth, suggesting that the BFHI had effectively increased breastfeeding initiation rates [12]. The most recent SADHS conducted in 2016 reported 32% of infants under the age of 6 months were exclusively breastfed, with breastfeeding being initiated within the hour within two-thirds (67%) of children and a mean duration of breastfeeding of 2.9 months as compared to 1.2 months in 1998 [13]. However, evidence [14–16] increasingly shows that maintaining optimal breastfeeding is related to factors in and outside the health system. In SA, mothers are discharged relatively early post-partum before breastfeeding is established [11, 17] and as a result, their exposure to breastfeeding support is limited, particularly during the first few vulnerable days when greater support is required. In the public sector, mothers may be discharged 6 h following normal vaginal delivery and 36 to 48 h following an uncomplicated caesarean delivery [17]. Consequently, a need arises for efforts to develop the social support system including the family and community continuum of care, which includes a mother’s broader support networks for continued and sustained breastfeeding efforts [11].

### Phasing out of the MBFI facility accreditation

In 2018, the revised WHO/UNICEF “Ten Steps to Successful Breastfeeding” were mandated as a standard of care for all healthcare facilities which provided maternal and child services, regardless of maternity bed availability. The process of Mother-Baby Friendly accreditation of facilities was then phased out [18]. In lieu of using facility accreditation as the primary outcome and driver of breastfeeding practice changes, the revised implementation guidelines of the Ten Steps emphasised integration of the guidelines into national policies, quality-improvement initiatives, and maternal and child health programmes [3]. The revised implementation guidelines highlight several challenges in BFHI implementation, such as the need for resources to maintain the processes of training, monitoring and assessment, a lack of sustainability of changes when a focus is placed on obtaining facility accreditation and the need for BFHI “champions” [3]. Similarly, in SA, challenges with MBFI implementation included the maintenance of Mother-Baby Friendly accreditation, the challenge of high staff turnover and shortages, which in turn influence maternity nurses to resort to older practises, and a perception that the MBFI is an additional burden rather than an essential component of routine maternal care [4, 11, 19].

In light of SA’s MBFI implementation challenges and the revised implementation guidelines’ strong emphasis on the integration of the Ten Steps into policy, the process of facility accreditation was phased out and the Ten Steps were mandated as a standard of care [18]. Facilities were strongly encouraged to monitor and support the implementation of the revised Ten Steps, and a WHO-compliant framework was created when SA adopted the 2018 revised BFHI guidelines to further guide implementation [20]. The framework aimed to support healthcare facilities in building a more robust programme and maintaining an improved quality of care over time. Included in the framework were implementation standards and practical steps to protect, promote and support breastfeeding in facilities providing maternity and newborn services.

### Problem statement

The negative impact of unexpected events, such as the COVID-19 pandemic, on breastfeeding in SA is highlighted in the SA Maternal, Perinatal, and Neonatal Health Policy addendum, “Challenges for maternal, perinatal, and neonatal services in South Africa” [21]. In December 2022, the SA Health Review 2022 [22], a review of various health and related indicators during and after the acute stage of the COVID-19 pandemic was published. The review indicated a decline in the prevalence of infants exclusively breastfed at the third dose of DTaP-IPV-Hib-HBV vaccination [22]. The review further suggests that there is a persistent trend of declining breastfeeding rates throughout the country, with the exception of the Gauteng and Western Cape provinces, where the rates have demonstrated relative stability [22]. The rates of exclusive breastfeeding following the third dose of DTaP-IPV-Hib-HBV vaccination are presented in Table 2. The data spans from 2017 prior to the onset of the COVID-19 pandemic until 2022, following the acute phase of the pandemic.

**Table 2:** Infant exclusively breastfed at DTaP-IPV-Hib-HBV 3rd dose rate (%) between 2017 and 2022

Province	2017/18	2018/19	2019/2020	2020/2021	2021/2022
Eastern Cape	46.7	50.0	48.2	45.2	43.2
Free State	53.8	53.8	53.6	46.4	44.0
Gauteng	47.4	46.1	45.7	45.2	46.6
KwaZulu-Natal	56.0	57.3	56.5	56.7	56.4
Limpopo	39.2	43.0	40.3	38.1	32.4
Mpumalanga	48.5	52.2	51.9	43.0	38.9
Northern Cape	56.0	55.3	55.9	52.7	49.0
North West	56.9	56.3	59.7	41.6	34.3
Western Cape	34.4	38.7	39.7	37.6	40.4
South Africa	47.8	49.5	48.8	45.9	44.6

Data from WebHDIS, extracted May 2022

In December 2021, SA provincial nutrition managers held a virtual meeting [23] to understand better the challenges associated with the declining exclusive breastfeeding rates (Table 2), and the implementation of the Ten Steps and monitoring activities. Insufficient breastfeeding support for mothers before their discharge from maternity facilities, abandonment of the implementation of the Ten Steps, and poor integration of monitoring activities, particularly at the facility level, were identified as challenges [23]. Furthermore, in some provinces, sessional doctors were not implementing evidence-based recommendations, while the COVID-19 pandemic contributed to a decline in routine services, and insufficient capacity building and monitoring in health facilities [23]. These challenges were reported as having negatively influenced exclusive breastfeeding rates.

## Methods

### Aim and objectives

In response to the declining exclusive breastfeeding rates and to support and protect breastfeeding in the country, the Child, Youth and School Health cluster of the NDoH, in collaboration with the WHO and UNICEF: SA hosted a two-day revitalisation workshop of the MBFI to address challenges and identify actions towards redress. The workshop took place in Johannesburg, SA, on the 29th and 30th of June 2022 and was facilitated by an academic (WL) affiliated with a South African university. The facilitator had expertise in workshop facilitation and a keen interest in the MBFI. As a global initiative, driven by the WHO and UNICEF, SA representatives of the organisations financially supported the meeting by covering travel and accommodation for participants as well as venue costs and facilitator fees.

Prior to the workshop, the following key objectives were identified:

1. To orientate participants with the MBFI implementation framework,
2. To identify key MBFI implementation bottlenecks,
3. To create an action plan for the revitalisation and implementation of the MBFI in all private and public facilities, which additionally mitigates the effects of the COVID-19 pandemic, and.
4. To agree on an accountability structure and platform for the integrated monitoring and reporting of MBFI implementation.

### Participants

The workshop was attended by representatives from the NDoH clusters for maternal and neonatal health, child and maternal health managers, provincial clinical managers, nutrition managers, District Clinical Specialist Teams (DCST), and other relevant program managers. Nursing service managers from various private groups who provided maternity services were requested to identify the most suitable person in their institution to be responsible for the MBFI. Invitations to the workshop from the NDoH requested that at least one representative from all nine provinces be in attendance, as well as at least one representative from the various private hospital groups invited. Four private hospital groups accepted the invitation, with three attending. A national coordinator of human milk banks, a specialist midwife and neonatal specialist, and clinical training specialists were among the private representatives. All nine provinces were in attendance and represented by two or more participants, with only one province having representatives who attended virtually.

Participants were seated at round tables and divided into one to two provinces, with the private hospital groups being considered a “province” and thus being seated together. Additionally, one table contained representatives from the WHO, UNICEF, and the International Baby Foods Action Network (IBFAN) Africa. Provincial representatives were grouped based on the belief that they would possess similar experiences. Similarly, the private hospital groups, known to implement institutional policies to maintain uniformity across their facilities, were seated together based on the belief that they would possess similar experiences. To promote collaboration among the grouped representatives, particularly during group activities, representatives from the WHO, UNICEF and IBFAN were seated together, separately from provincial and private representatives.

### Processes

The NDoH established an agenda through internal processes, considering the workshop’s objectives, identified priorities, and policy guidelines. Although the agenda offered a structured framework, an effort was put forth to foster collaboration and encourage the involvement of the various provincial and private representatives. The following section offers a brief description of the process and sequence of the workshop’s activities.

#### Day one

The first day began with a group work session to identify participant expectations. The NDoH then presented the background and purpose of the meeting, an overview of the MBFI in SA and a review of the MBFI implementation framework. This facilitated a mutual understanding of the initiative as different participants, depending on their roles in the health sector, were likely to have varying levels of familiarity with its history and implementation. To provide insight into other countries’ experiences, a representative of the UNICEF: Eastern and Southern Africa Regional Office (ESARO) presented a session on the experiences of other countries in implementing the 2018 revised UNICEF/BFHI guidelines. This was followed by an Infant and Young Child Feeding (IYCF) consultant, who presented an update on the WHO/UNICEF BFHI tools and materials. Presentation materials were shared with workshop participants via Google Drive.

Following the presentations, a second group work session, facilitated by WL, was held in which participants worked in smaller groups of six to eight people to identify the strengths and weaknesses of MBFI implementation. As part of this session, participants were asked to organise the revised BFHI Ten Steps from most challenging to least challenging to implement. Thereafter, a discussion of the potential models for MBFI monitoring, accountability, and reporting was held based on the participant’s feedback. Monitoring facility adherence to the Ten Steps is a critical component of the WHO/UNICEF revised guidelines [3], it was therefore essential that participants actively participated in identifying solutions for monitoring, accountability, and reporting applicable to the SA context.

#### Day two

On the second day, participants worked in provincial groups to create contextual action plans that included at least the three most challenging steps of the BFHI to implement within their given context. Following the workshop, provincial representatives were given an additional two weeks to collaborate with their teams, expand on their preliminary workshop action plans, and present an action structure using the NDoH’s action plan template. These action plans were uploaded to a national repository to serve as baseline data for future analysis. To conclude the workshop, participants were asked to restructure the information they had shared and gathered during the workshop and present it as a single idea that they found most impactful.

## Findings

The subsequent section provides a narrative report of the outcomes of the presentations and group sessions, as facilitated by the academic (WL). Permission to report on the structure and outcome of the workshop was obtained from the NDoH.

### Presentations

#### NDoH: an overview of the MBFI monitoring and evaluation strategies in SA

The NDoH provided an overview of the status and progress of implementing the WHO guidance and recommendations for establishing integrated monitoring and evaluation strategies. Progress made in MBFI implementation includes the incorporation of indicators of early breastfeeding initiation in the national indicator data set (NIDS). Following the 2011 Tshwane Declaration, the 2013 National IYCF policy, indicated that “all health facilities with maternity beds should implement the MBFI” [24]. Subsequently, as of the 2013/14 FY, the District Health Information System (DHIS) included the infant exclusively breastfed at DTaP-IPV-HiB-HBV 3rd dose rate (administered at 14 weeks) indicator, which enabled the NDoH to measure progress towards achieving the six-month exclusive breastfeeding rate. The South African Demographic and Health Survey was used to collect data from the six-month indicator in a typically small sample [13] and included the breastfeeding indicator at 14 weeks, this allowed for the identification of areas where intervention was required.

Policy alignment was discussed, and at the time of the workshop, the comprehensive alignment of MBFI in the revised IYCF policy guidelines was being finalised. The NDoH stated that alignment with the Maternal, Perinatal, and Neonatal Health (MPNH) policy of 2021 [25] was complete, and that exploration into how to incorporate some aspects of the MBFI in the Ideal Hospital framework [26] was underway. Furthermore, integrated clinical service management components such as clinical guidelines and protocols have been incorporated into the Ideal Clinic realisation framework, including monitoring whether clinical guidelines and protocols are available, staff have received training on their use, and if they are being applied appropriately [27]. Additionally, the maternity case record includes a section where infant feeding is discussed, and the quality improvement plan addresses all areas and is signed and updated quarterly [27]. Since the implementation of the IYCF policy, facilities have access to national guidelines on priority health conditions. The policy states that at least 50% of nurses are trained in the MBFI, although the global criteria are 80% [24].

#### UNICEF: ESARO: an overview of BFHI implementation in other African countries

The maternal nutrition landscape analysis conducted in the East and Southern Africa region shows that challenges persist in the implementation of maternal, infant and young child nutrition intervention, particularly low coverage in the implementation of nutrition interventions during antenatal care [28] despite WHO guidelines of “antenatal care for a positive pregnancy experience” [3]. The UNICEF: ESARO representative highlighted the significance of the healthcare system as a delivery platform for interventions in maternal, infant, and young child nutrition and noted that vertical BFHI implementation strategies proved to be barriers to the initiatives’ implementation in African countries, whereas leadership and coordination at all levels were crucial success factors.

Emergency Obstetric and Newborn Care (EmONC) in Zimbabwe and Quality Improvement for Maternal and Neonatal Health (MNH) in Ghana were used as entry points for the BFHI. Both countries developed standards that included the Ten Steps, with Zimbabwe taking an integration approach, incorporating the BFHI into other programs such as Maternal and Newborn Health and Nutrition and promoting joint mentorship, monitoring, advocacy, and training, and Ghana emphasising stakeholder involvement, tracking tools, and scorecards.

An important lesson learnt from these countries is that functional Newborn/Breastfeeding subcommittees at a national and sub-national level are excellent platforms for maternal and newborn health and breastfeeding coordination and collaboration. Furthermore, these countries show that leveraging existing systems and interventions facilitates buy-in from key stakeholders. The integration of Maternal Neonatal Health and Quality Improvement (MNH-QI) and the BFHI, on the other hand, is a gradual process that countries such as SA must define within their given context. Technical and financial support for advocacy, capacity development of healthcare providers, and monitoring by national and sub-national teams are required for such a process to be sustainable in South Africa and other countries worldwide [29, 30].

### Group sessions: participant feedback

#### Session one: workshop expectations

Round table discussions were held to identify participant expectations before the start of the workshop. Participant expectations prior to the workshop included strengthening the mother-baby pair, family integration and community systems, and ensuring that breastfeeding became a standard of care in all facilities. Participants inquired about integrating MBFI with existing programs, including practising the initiative while ensuring COVID-19 safety, and how they should review the monitoring process when a lack of interest in MBFI implementation was evident without accreditation. A strong expectation was that of developing an MBFI monitoring framework, integration approach and action framework. Participants emphasised the importance of MBFI strategies being context-specific and involving communities and all involved stakeholders. Other expectations of wanting to know how to strengthen antenatal care, the first golden hour and postnatal care, and addressing sustainability, networking, and international support between public and private sectors were voiced.

#### Session two: identifying bottlenecks and proposed actions

In small groups of six to eight participants, the following three questions were asked; (1) prioritise the ten steps in your context from the most challenging to the best performer, (2) unpack the bottlenecks, and (3) identify action plan to address bottlenecks and in addition identify them as: M–maternal factors, B–baby factors, F–friendly (facility factors) and/or I–initiative (policies and other documents/ regulations).

#### Bottlenecks based on the most to least challenging steps of the BFHI to implement

Participants identified Step ten “coordinating discharge so that parents and their infants have timely access to ongoing support and care” of the revised Ten Steps [3] as the most challenging to implement. Several factors were highlighted to contribute to the difficulties encountered in implementing this step. These factors included a scarcity of community resources dedicated to promoting breastfeeding, high levels of mobility within communities, insufficient community engagement, a lack of social support, inadequate connections between delivery facilities and post-natal units at the community level, cultural beliefs, and insufficient coverage of catchment areas by community health workers who lack the necessary training on MBFI.

Step ten was followed by step five “supporting mothers to initiate and maintain breastfeeding and manage common difficulties”. The reported challenges of this step included inadequate infrastructure to support breastfeeding, such as lodger facilities; distance between labour and neonatal wards; facilities which have high birth rates may lack sufficient staff to provide support; staff and resource shortages limited the availability of support for mothers; and inadequate healthcare worker support to mothers. Step two “ensuring that staff have sufficient knowledge, competence and skills to support breastfeeding” and step three “discussing the importance and management of breastfeeding with pregnant women and their families” [3] were identified as the next most challenging steps to implement. Step two is considered a critical management procedure and challenges reported included a lack of in-service training for medical doctors who may still use old practices, poor screening of trainers, poor training methods, poor training of caregivers and outreach teams, an unwillingness to attend training for 20 h due to staff shortages, a lack of mentorship and the negative impact of COVID-19 on training. Challenges involved in the implementation of step three included inadequate information to mothers due to health system factors and staff shortages, a lack of staff confidence in providing antenatal education and counselling and high rates of teenage pregnancy.

On the contrary, step seven, “enabling mothers and their infants to remain together and to practice rooming-in 24 hours a day” was identified as the easiest to implement. This step is regarded as less resource-intensive compared to the previously mentioned steps. This is because the largest challenge encountered in implementation was that of providing adequate space for mothers and infants. Therefore, significant investments in additional material and human resources were not necessary.

#### Proposed actions

Participants voiced that since the phasing out of MBFI accreditation, some facilities were able to maintain good breastfeeding practices, while others struggled. Gaps in monitoring and reporting of MBFI implementation were emphasised as being a key implementation bottleneck. Potential actions to improve implementation, as identified by participants, centred around strengthening capacity building, and mentorship which included the use of existing assessors for training staff, monitoring as part of district peer review, the integration of accountability measures in facility chief executive officer (CEO) key performance areas, and the issuing of certification to acknowledge compliant facilities. Whilst participants identified recertification as a potential action plan to improve the implementation of the Ten Steps, a culture of being “mother-baby friendly” and improved sustainability was also emphasised through the integration and strengthening of capacity building, monitoring, and accountability measures.

#### Session three: take home messages

Concluding the workshop, participants were asked to restructure the information shared and received and present it as one idea they found most impactful. Participants highlighted a need to strengthen or resuscitate the MBFI within the country, adding that there needed to be collaboration, revitalisation, and resuscitation of already existing strategies. A unanimous agreement among participants was that it was important to document or publish the SA MBFI findings to inform a review of the global recommendations, particularly the strategies and guidelines of the WHO and UNICEF.

## Discussion

### SA challenges in implementing the revised ten steps

Implementation challenges of the MBFI and the revised Ten Steps are evident in SA literature [4, 11, 14, 15, 18, 19, 21] and further confirmed by workshop participants. The initial rollout of the MBFI was reported to be sluggish [6, 11] prior to the 2011 adoption of the Tshwane Declaration of Support for Breastfeeding [9]. However, rates of facility accreditation and exclusive breastfeeding varied significantly across provinces with Gauteng and the Western Cape consistently performing better [11]. Moreover, there was criticism that the initiative functioned in a state of isolation, lacking integration into the broader public health system [11]. This was accompanied by a lack of adequate resources and training, as well as inadequate management and mentoring [11].

BFHI targets and accreditation motivate hospital performance [11] but have not been accompanied by sustainability plans. As a result, it is difficult to assess whether designated facilities have continued to meet the MBFI criteria and thus contribute to improving maternal, child and nutrition outcomes in the country. It is reported that in some provinces, districts, and facilities, MBFI training and assessment costs prevented full implementation of the initiative [11, 14]. Additionally, the HIV epidemic contributed to a “spill-over” of messages to avoid all breastfeeding even among HIV-negative or unknown-status populations and influenced public opinion on breastfeeding [11, 14]. Moreover, a high degree of staff rotation is commonly reported at hospitals and continues to be an implementation challenge [11, 19]. The MBFI contributes significantly to the protection, promotion, and support of breastfeeding in SA. However, as a resource-intensive and often vertical and nutrition-driven strategy, a resultant lack of ownership and ineffective implementation and sustainability is observed [11].

### Revitalising the MBFI in SA: the way forward

Moving towards revitalisation raises the question of what is meant by revitalisation. Realistic activities with timelines must be established, which revise and re-establish the coordination mechanisms of existing platforms. While capacity building of health workers also needs to be revived to support the implementation of clinical practice with further community engagement in Protection, Promotion and Support (PPS), side-by-side campaigns and community outreaches. Consequently, it is important that the action plans submitted by each province be reviewed and each province identifies individuals or teams who can take responsibility for the initiative in their respective contexts. Facility policies should be revised to align with the framework and the MBFI should be a standing agenda point on the Maternal, Child and Women’s Health and Neonatal meetings.

#### Monitoring of the MBFI revitalisation and implementation

An improved integrative strategy must be used to accurately monitor the Ten Steps implementation and revitalise the MBFI. Therefore, as recommended by the WHO/UNICEF revised guideline [3] routinely collected sentinel indicators, such as the early breastfeeding initiation and exclusive breastfeeding at the DTaP-IPV-HiB-HBV 3rd dose rate (around 14 weeks), should be used as a proxy indicator to track MBFI implementation. Annual self-assessment tools should be integrated with existing quality improvement and monitoring systems or initiatives [3], with an exploration of the Ideal district hospital initiative [26]. Additionally, healthcare facilities need to have an ongoing monitoring system in place, this should include quarterly or biannual facility support and monitoring visits from provincial and district officials. A national random selection of facilities should also be conducted annually, and monitoring reports should be submitted to the NDoH.

Reporting should be conducted using integrated facility action plans available for annual self-appraisals. Following the workshop, approved annual reports were submitted using the NDoH’s developed template, and platforms for reporting and sharing updates at annual, biannual, and quarterly maternal, child and women’s health (MCWH)/child youth, and school health and nutrition meetings were strongly advised. In the meantime, it should be noted that the WHO BFHI monitoring manual is being developed.

#### Maternal discharge surveys

In addition to confidential written, oral, and SMS feedback, maternal discharge surveys, such as exit interviews with approximately 20 mothers per cohort, can be conducted. Based on feedback from the workshop, the role of operational managers (OPMs) or quality assurance managers should be investigated further to facilitate discharge surveys. OPM and DCST supervision tools can be used, and the MBFI should be included in OPM’s key performance areas and staff performance agreements.

#### Training

Healthcare facilities should adopt various strategies for training, and the NDoH should explore incorporating training into the Knowledge Hub. Collaboration with universities to establish an online training platform can be explored further. Training packages for medical schools or nursing colleges should be aligned with current content within the university curriculum of healthcare students to introduce a breastfeeding course in pre-service. Existing training programmes should be scrutinised, and the breastfeeding training package should be shared and integrated into existing training. To develop integrated training, the Child, Youth, and School Health cluster should consult with the NDoH nursing directorate. Additionally, the integration of training in other curricula, such as managing small and sick newborns (MSSN) should be considered.

It is crucial to involve the private sector and repackage training while exploring various methods of training, such as the incorporation of the MBFI in existing training, the presentation of webinars and communication of breastfeeding as part of antenatal topics. Training on the Regulations relating to Foodstuffs for Infants and Young Children is also critical in complying with the International Code of Marketing of Breastmilk Substitutes. At the same time, the training of breastfeeding counsellors in communities should further be prioritised, together with the scaling up of social mobilisation of breastfeeding in communities [3]. In this regard, the Baby-Friendly Community Initiative [31] and the role it could play in SA should be explored further.

### Recommendations

The NDoH recommended a self-appraisal situational analysis of all facilities that previously obtained MBFI accreditation. A baseline assessment or situational analysis of current MBFI practices is to be performed. Sensitisation workshops must be presented and feedback on MBFI implementation and new monitoring strategies must be provided. Stakeholder engagement, role clarification and ownership of the MBFI implementation and monitoring processes are needed. While coordination mechanisms need to be revised and re-established, facility self-appraisal should precede formal provincial monitoring.

### Strengths and limitations

The MBFI revitalisation workshop, primarily driven by the NDoH in response to declining breastfeeding rates, presents both strengths and limitations. The workshop’s inherent vertical nature is a limitation in the revitalisation efforts of the MBFI. The effort to open dialogue and encourage a shift towards a more contextually appropriate and collaborative approach was a key strength of the workshop that attempted to mitigate this. Participants were actively encouraged to share their views, express their concerns, and offer recommendations; their feedback will be crucial in determining the direction of the revitalisation efforts.

On the other hand, a limitation of this paper pertains to the absence of primary data, such as direct participant quotes. Additionally, there is a need to follow up with participants to ensure that action plans were submitted. Nonetheless, the dissemination of data on the MBFI is a significant strength. This dissemination could help to promote the MBFI and its revitalisation efforts, as well as serve as a valuable resource for both the initiative and its stakeholders, as well as other countries implementing the BFHI.

## Conclusion

Previous lessons learned and recommendations regarding the MBFI implementation in SA included: when the MBFI is integrated with newborn care, particularly KMC, implementation is propelled, facilities with higher numbers of deliveries should be prioritised, while strong community support and active social mobilisation interventions enhance MBFI effectiveness. Additionally, the MBFI should be included as a key performance indicator in the broader health information management system at district levels and should go beyond increasing breastfeeding rates alone. Finally, breastfeeding promotion has been and continues to be, a South African priority action as it contributes to achieving the 2030 National Development Plan goals [32].

The MBFI has evolved over many years since its introduction and SA has great strength to further build on the initiative and its successes. With a need to revitalise pathways and strengthen community engagement, the MBFI should broaden its focus beyond hospitals. To move the MBFI forward, the country requires mechanisms to help realise this and to finalise an implementation plan which reflects its importance and focuses on its key aspects, rather than nice-to-have features.

## Data Availability

Not applicable.

## Abbreviations

BFHI: Baby-Friendly Hospital Initiative
CEO: Chief Executive Officer
DCST: District Clinical Specialist Teams
DHIS: District Health Information System
EmONC: Emergency Obstetric and Newborn Care
ESARO: Eastern and Southern Africa Regional Office
FY: Financial Year
IBFAN: International Baby Foods Action Network
IYCF: Infant and Young Child Feeding
KMC: Kangaroo Mother Care
MBFI: Mother-Baby Friendly Initiative
MCWH: Maternal, Child and Women’s Health
MNH-QI: Maternal Neonatal Health and Quality Improvement
MNH: Maternal and Neonatal Health
MPNH: Maternal, Perinatal, and Neonatal Health
MSSN: Managing of Small and Sick Newborns
NDoH: National Department of Health
NIDS: National Indicator Data Set
OPM: Operational Manager
PMTCT: Prevention of Mother-to-Child Transmission
PPS: Protection, Promotion and Support
SA: South Africa
SADHS: SA Demographic and Health Survey
UNICEF: United Nations Children’s Fund
WHO: World Health Organization
